# Motor asymmetry in Parkinson’s disease: Diagnostic thresholds based on clinical scores and DaTSCAN imaging

**DOI:** 10.1016/j.prdoa.2025.100350

**Published:** 2025-05-19

**Authors:** Philippe Voruz, Julie Péron

**Affiliations:** a Clinical and Experimental Neuropsychology Laboratory, Department of Psychology and Swiss Centre for Affective Sciences, University of Geneva, Geneva, Switzerland; b Department of Neurosurgery, University Hospitals of Geneva, Geneva, Switzerland; c Geospatial Molecular Epidemiology Group, Laboratory for Biological Geochemistry, School of Architecture, Civil and Environmental Engineering, Ecole Polytechnique Fédérale de Lausanne, Lausanne, Switzerland; d Department of Neurology, University Hospitals of Geneva, Geneva, Switzerland

**Keywords:** Motor Asymmetry, Parkinson’s Disease, DaTSCAN, MDS-UPDRS III, ROC Curve, Diagnostic Thresholds

## Abstract

Motor asymmetry is a key feature of Parkinson’s disease that can influence disease progression and patient outcomes. However, there is currently no consensus on the best method or clear thresholds to determine motor asymmetry, which limits the ability to standardize its assessment across studies. This work aimed to determine which motor asymmetry score − standardized or non-standardized − best predicts dopaminergic damage in the putamen, as measured by DaTSCAN, and to establish clear thresholds for distinguishing symmetric from asymmetric profiles. Data from the Parkinson’s Progression Markers Initiative were utilized for the Receiver Operating Characteristic curve analysis. Both motor asymmetry scores significantly predicted dopaminergic asymmetry, with the non-standardized score showing a slightly higher predictive power (area under the curve [AUC] = 0.621, p < 0.001) compared to the standardized score (AUC = 0.569, p = 0.018). For the non-standardized score, a cut-off of ± 2.50 yielded a sensitivity of 0.874 and specificity of 0.783. In contrast, the standardized score achieved a cut-off of ± 0.188, resulting in a sensitivity of 0.908 and specificity of 0.823. These findings underscore the importance of motor asymmetry in clinical evaluations of Parkinson’s disease and provide a foundation for further research aimed at refining diagnostic thresholds.

## Highlights

• Cut-off for symmetrical Parkinson’s disease based on motor scores.
• Non-standardised asymmetry with better power.
• Parkinson’s disease risk factor based on motor symptoms.

## 1 Introduction

Motor asymmetry is a hallmark feature of Parkinson’s disease (PD), often evident in the early stages of the condition [1]. Importantly, motor asymmetry, and even the specific side of the body predominantly affected (right vs. left), has been shown to be a predictor of the progression of both motor [2] and non-motor symptoms in PD [3], influencing disease trajectory and patient outcomes [2,3]. Given its prognostic value, motor asymmetry should be systematically considered in future studies. However, there is currently no consensus on the best method or clear thresholds to determine motor asymmetry, which limits the ability to standardize its assessment across studies. This is increasingly important as recent models, such as the α-synuclein origin and connectome model (SOC Model) of PD [[4], [5], [6]], incorporate asymmetry and symmetry to distinguish phenotypes. However, without a clinical cut-off for motor symptoms, empirical assessment and clinical management remain limited. Over the past decades, various methods have been developed to quantify motor asymmetry, using both clinical symptom evaluations [3,[7], [8], [9], [10], [11], [12], [13]] and neuroimaging data [14,15], with the goal of improving disease understanding and diagnostic approaches. These methods generally fall into two main categories: those based on clinical assessment of motor symptoms, and those based on imaging techniques such as DaTSCAN, which measures the asymmetry of nigrostriatal denervation.

In clinical evaluations, motor symptom asymmetry is often measured using lateralized items from part III of the Unified Parkinson’s Disease Rating Scale (MDS-UPDRS III [16]). Several equations have been proposed to quantify this asymmetry, including simple subtractions between scores for the left and right sides of the body, as well as more complex asymmetry indices that account for the magnitude of the difference [12,13]. However, while these methods are widely used, they do not always provide a clear distinction between patients with symmetric and asymmetric motor symptoms, due to the lack of established thresholds for this classification.

On the other hand, the use of DaTSCAN, a neuroimaging technique that quantifies dopaminergic denervation, has introduced new possibilities for assessing asymmetry in PD. Asymmetry indices for the striatum, typically calculated by comparing the left and right putamen, have enabled researchers to define specific thresholds, such as a 20 % asymmetry cutoff, to differentiate between patients with symmetric and asymmetric disease profiles [14,15]. However, few studies have directly compared motor symptom asymmetry with DaTSCAN asymmetry, and a consistent relationship between clinical asymmetry and nigrostriatal denervation has not been established for all patients [14].

Given the lack of consensus on the best methods for measuring motor asymmetry and the absence of clear diagnostic thresholds for motor symptoms, comparing findings across studies remains challenging. This underscores the need for statistical thresholds to reliably distinguish patients with symmetric and asymmetric motor symptoms, integrating both motor scores and neuroimaging data.

The aim of this study is to identify which motor asymmetry score, standardized or non-standardized, best predicts asymmetry as measured by DaTSCAN in patients with PD, and to establish clear thresholds to distinguish symmetric from asymmetric profiles. To achieve this, we will use data from the Parkinson’s Progression Markers Initiative (PPMI) and conduct Receiver Operating Characteristic (ROC) curve analysis to evaluate the diagnostic accuracy of motor asymmetry scores and determine the optimal threshold for patient classification.

## 2 Methodology

### 2.1 Participants

The data used in this article were obtained from the PPMI an observational, international, multicenter study focused on identifying biomarkers of Parkinson's disease progression [17]. Data used in the preparation of this article were obtained on [2024–09–25] from the PPMI database (https://www.ppmi-info.org/access-data-specimens/download-data), RRID:SCR_006431. Clinical trial number: not applicable. For up-to-date information on the study, visit http://www.ppmi-info.org.

The analysis in this paper is based on baseline measurements. Retrospectively, neuroimaging and motor symptom data were extracted for 402 patients with sporadic PD. The baseline inclusion criteria for participants with PD aged over 30 were as follows: 1) newly diagnosed PD (within two years); 2) no prior treatment with PD medication; 3) and to have either at least two motor symptoms: i) resting tremor, ii) bradykinesia, and/or iii) rigidity. Mandatory to have either resting tremor or bradykinesia or a single asymmetric resting tremor or asymmetric bradykinesia (measured with MDS-UPDRS III); 4) imaging confirmation of a dopamine transporter deficit. Moreover, clinical information’s concerning patients are provided within Table 1. Written informed consent for research was obtained from all individuals participating in the PPMI study.

**Table 1:** Socio-demographical and clinical information of the Parkinson’s disease patients included in the study.

	**Whole group** **(n = 402)**
**Age (in years)**	61.97 ± 9.50
**Gender (M/F)**	260/142
**Disease duration (inclusion criteria)**	< 2 years
**Handedness**	R: 84.90 %; L: 13.40 %; Amb: 1.70 %
**DaTSCAN asymmetry score**	0.02 ± 0.22
**MDS-UPDRS III Total score**	21.33 ± 9.18
**MDS-UPDRS III Lateralized items (standardized)**	0.98 ± 0.69
**MDS-UPDRS III Lateralized items (non-standard standardized)**	−0.22 ± 6.57

Legend. DaT-SCAN asymmetry score : ioflupane 123I-FP-CIT dopamine transporter (DaT) SPECT) with standardized formula used: (Left putamen − Right putamen) / (Left putamen + Right putamen); F: Amb: Ambidextre; F: Female; L:Left; R: Right.

*Ethics.* This study was conducted in accordance with the Declaration of Helsinki and the Good Clinical Practice guidelines after approval of the local ethics committees of the participating sites. Written informed consent for research was obtained from all individuals participating in the study.

*Data availability.* All data is extracted from the PPMI funded by the Michael J. Fox Foundation for Parkinson's Research and funding partners.

### 2.2 Neuroimaging asymmetry measure

In this study, we extracted data from DaTSCAN (ioflupane 123I-FP-CIT dopamine transporter (DaT) SPECT) nortropane single photon emission computed tomography], which was acquired at PPMI imaging centers according to the PPMI baseline imaging protocol. Following validated methodologies from previous studies [14,15], we calculated a standardized asymmetry/symmetry score for dopaminergic loss in the putamen of the left and right hemispheres. The standardized formula used was: (Left putamen − Right putamen) / (Left putamen + Right putamen). Based on criteria validated in earlier studies, a cut-off of ± 20 % was applied to classify participants as having either asymmetric or symmetric PD pathology.

To classify patients as having either symmetric or asymmetric dopaminergic degeneration, we applied a cut-off of ± 20 %, a threshold supported by earlier research and clinical convention. This value is based on the premise that normal physiological asymmetry typically falls within a ± 10–15 % range; therefore, deviations beyond ± 20 % are more likely to reflect pathologically significant differences. Prior studies using similar SPECT imaging modalities have adopted this cut-off to distinguish between symmetrical and asymmetrical PD phenotypes, with the threshold shown to correspond to clinical features such as lateralized motor symptom severity and disease progression patterns [14,15]. The 20 % threshold thus balances sensitivity to detect relevant asymmetry while minimizing the misclassification of normal interhemispheric variation.

### 2.3 Motor symptom asymmetry measure

To assess motor symptoms, we extracted data from the MDS-UPDRS III (Fahn et al., 1967). To evaluate motor asymmetry, we focused on items which assess asymmetric motor functions (left and right sides). Given the lack of consensus on how to measure asymmetry and the lack of a cut-off to determine a symmetrical form of PD pathology based on motor symptoms, we applied two main formulas as described in the literature: i) a non-standardized motor asymmetry score [10]: (Left MDS-UPDRS III motor symptoms − Right MDS-UPDRS III motor symptoms); and ii) a standardized score [[12], [13], [14]]: (Left MDS-UPDRS III motor symptoms − Right MDS-UPDRS III motor symptoms) / (Left MDS-UPDRS III motor symptoms + Right MDS-UPDRS III motor symptoms).

### 2.4 Statistical analysis

To determine which motor asymmetry score best predicts asymmetry or symmetry as measured by DaTSCAN, and to statistically establish thresholds for distinguishing between patients with asymmetric motor symptoms and those with symmetric motor symptoms, we conducted ROC Curve analysis. This method is a robust statistical tool for evaluating diagnostic accuracy, providing a clear trade-off between sensitivity and specificity, while enabling performance assessment across various thresholds. In this analysis, the dependent variable was the dichotomized DaTSCAN scores at baseline, with cut-offs used to classify individuals as having symmetric PD (coded as 0) or asymmetric PD (coded as 1). The independent variables included two motor asymmetry scores: i) the non-standardized score and ii) the standardized score. Finally, post-hoc ROC analyses were performed adding gender and age as variables of interest.

## 3 Results

**Fig. 1:**
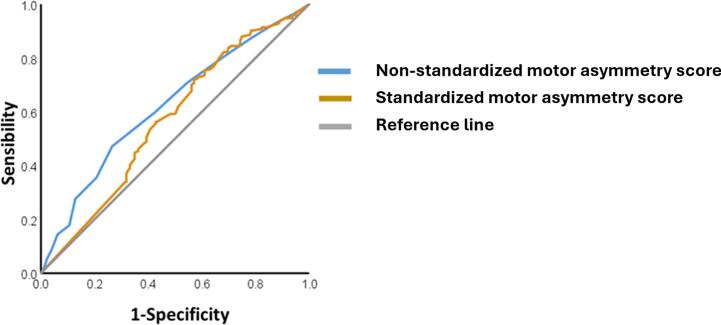
Receiver operating characteristic (roc) curve analysis of motor asymmetry scores predicting dopaminergic damage in the putamen.

The ROC analysis, using asymmetric/symmetric dichotomization based on DaTSCAN results as the dependent variable, demonstrated that both the standardized and non-standardized motor asymmetry scores predicted dopaminergic damage in the putamen (asymmetric/symmetric score; see Fig. 1). Specifically, the analysis revealed that the non-standardized score was significant, with an area under the curve (AUC) of 0.621 (p < 0.001; 95 % CI [0.565, 0.676]), while the standardized score also reached significance, covering an area of 0.569 (p = 0.018; 95 % CI [0.513, 0.625]). These results indicate that the non-standardized motor score has slightly better predictive power for distinguishing symmetric and asymmetric dopamine loss in the putamen.

The main associations between motor asymmetry scores and dopaminergic asymmetry remained stable and statistically significant after adjusting for gender and age.

Based on the optimal cut-off values for sensitivity and specificity (0.80 for both), as determined statistically by Unal [18], we observe that for the non-standardized score, the ideal cut-off is approximately ± 2.50, yielding a sensitivity of 0.874 and specificity of 0.783. For the standardized score, the optimal cut-off is approximately ± 0.188, with a sensitivity of 0.908 and specificity of 0.823.

## 4 Discussion

The findings of this study provide new insights into the predictive value of motor asymmetry scores for distinguishing between symmetric and asymmetric dopaminergic degeneration in PD, as measured by DaTSCAN. Both standardized and non-standardized motor asymmetry scores significantly predicted asymmetry in putaminal dopaminergic loss, though the non-standardized score showed slightly better predictive power. These results have important implications for improving diagnostic accuracy and establishing thresholds for motor asymmetry in PD research and clinical practice.

The ROC analysis showed that the non-standardized motor asymmetry score achieved an AUC of 0.621, compared to 0.569 for the standardized score. Although both scores reached statistical significance, the higher AUC associated with the non-standardized score suggests it may serve as a more reliable predictor of dopaminergic asymmetry in the putamen. This aligns with previous findings indicating that simple, non-normalized motor assessments can, in some cases, yield clearer clinical differentiation in patients with PD [7,19]. The superior sensitivity and specificity of the non-standardized score further support its potential utility as a practical tool for clinical classification. However, despite statistical significance, the observed AUC values reflect only modest discriminative performance. Given that the goal of this study is to inform clinical or research stratification based on asymmetry, it is important to emphasize that these scores should be interpreted cautiously and ideally considered alongside complementary clinical or neuroimaging measures.

Interestingly, although the non-standardized score demonstrated slightly better overall performance, both scoring methods yielded comparable sensitivity and specificity. Specifically, the non-standardized score, using a cut-off of ± 2.50, achieved a sensitivity of 0.874 and a specificity of 0.783, while the standardized score, with a cut-off of ± 0.188, reached a sensitivity of 0.908 and a specificity of 0.823. These high values suggest that both approaches are effective in distinguishing between symmetric and asymmetric motor symptoms. However, the choice between them may depend on the specific clinical or research context. The standardized score, which adjusts for the total magnitude of motor symptoms, may be particularly useful in studies aiming to control for overall disease severity. That said, one potential drawback of standardization is that it may disproportionately penalize individuals with low total motor scores, where even small absolute side differences, often clinically relevant in early-stage or mild disease are minimized or masked by normalization. Conversely, in patients with a high unilateral burden, normalization may obscure meaningful asymmetries by compressing the score range and reducing between-subject variability. These considerations suggest that while standardization ensures consistency across individuals, it may not always preserve the clinical relevance of asymmetry across the full spectrum of PD severity.

As previously noted in the introduction, motor asymmetry has been shown to predict not only the progression of motor symptoms but also non-motor symptoms in PD. This suggests that asymmetry is a fundamental feature of disease progression, which should be systematically considered in clinical studies and treatment plans. Moreover, the identification of cut-off thresholds for motor asymmetry scores − particularly the non-standardized score can help guide clinical decisions regarding patient classification. Establishing a threshold of ± 2.50 for non-standardized scores, for example, could serve as a reliable marker for distinguishing patients with symmetric versus asymmetric motor symptoms, facilitating early interventions, stratified treatment approaches or comparisons between previous studies within meta*-*analyses for instance.

While this study provides valuable insights, several limitations should be considered. First, in the present cohort, cerebral asymmetry was assessed using DAT-SPECT imaging, a technique with inherent limitations, particularly in spatial resolution that may have led to an underestimation of asymmetry. Future studies should consider employing PET imaging, which offers superior resolution, to enhance the accuracy and sensitivity of asymmetry detection. Second, PPMI is a multicentric cohort study. This approach, while offering significant advantages in terms of sample size, may be subject to methodological limitations, including potential biases arising from the involvement of multiple independent evaluators. Third, the AUC values for both the standardized and non-standardized scores, while significant, suggest only moderate predictive power. An AUC of 0.621 for the non-standardized score, for instance, indicates room for improvement in the predictive models used. This could be due to the inherent variability in both motor symptoms and neuroimaging data, as well as the complex relationship between clinical symptoms and dopaminergic degeneration. As noted by [14], a minority of patients show ipsilateral motor symptoms relative to putaminal denervation, which may partially explain the moderate AUC values observed. Furthermore, although the DaTSCAN data utilized in this study assesses dopaminergic denervation in the striatum, it does not consider asymmetries in other important brain structures that could also influence motor symptoms. For example, the substantia nigra, globus pallidus, subthalamic nucleus, and even the cerebellum may significantly contribute to the overall motor function and asymmetry seen in patients with PD. Finally, since this analysis was based on data from the PPMI cohort and this is only carried out on patients at the time of diagnosis. A longitudinal analysis would be of interest to see how the AUC changes over time. Therefore, the results may differ from other PD populations in terms of demographic and clinical characteristics, replication of these results in other datasets would be beneficial.

Future research should aim to refine and validate motor asymmetry thresholds by incorporating additional clinical variables, such as non-motor symptoms and cognitive decline. This study is based exclusively on baseline data from the PPMI cohort, which, while valuable for identifying initial patterns, limits the ability to assess prognosis or disease progression. Given that motor asymmetry may influence both disease trajectory and subtype classification in PD, it is crucial to evaluate whether the identified asymmetry scores and thresholds can predict longitudinal outcomes. Follow-up studies using the PPMI cohort or other longitudinal datasets are essential to confirm the prognostic utility of these measures. Additionally, the integration of advanced imaging techniques, such as functional MRI or PET scans, alongside DaTSCAN could enhance the precision of asymmetry assessments and provide deeper insights into the neural mechanisms underlying motor asymmetry in PD.

## 5 Conclusion

In conclusion, this study demonstrates that both standardized and non-standardized motor asymmetry scores are significant predictors of dopaminergic asymmetry in PD, with the non-standardized score showing slightly better predictive power. These findings provide a foundation for future work to establish clear diagnostic thresholds based on motor scores, allowing for improved comparisons across studies in PD research.

## CRediT authorship contribution statement

**Philippe Voruz:** Writing – original draft, Methodology, Formal analysis, Data curation, Conceptualization. **Julie Péron:** Conceptualization, Formal analysis, Funding acquisition, Methodology, Writing – review & editing.

## Ethics approval and consent to participate

The PPMI study is registered with ClinicalTrials.gov (NCT01141023). Each participating PPMI site received approval from an ethical standards committee on human experimentation before study initiation. This study was conducted in accordance with the Declaration of Helsinki and the Good Clinical Practice guidelines after approval of the local ethics committees of the participating sites. Written informed consent for research was obtained from all individuals participating in the study.

## Data availability

All data is extracted from the Parkinson's Progression Marker Initiative funded by the Michael J. Fox Foundation for Parkinson's Research and funding partners.

## Declaration of competing interest

The authors declare that they have no known competing financial interests or personal relationships that could have appeared to influence the work reported in this paper.
